# Staff and voice hearer perspectives on Hearing Voices Groups in the NHS: a mixed-methods cross-sectional survey

**DOI:** 10.3389/fpsyg.2025.1583370

**Published:** 2025-07-04

**Authors:** Alison Branitsky, Anthony P. Morrison, Eleanor Longden, Sandra Bucci, Filippo Varese

**Affiliations:** ^1^Division of Psychology and Mental Health, School of Health Sciences, Faculty of Biology, Medicine and Health, Manchester Academic Health Science Centre, The University of Manchester, Manchester, United Kingdom; ^2^Psychosis Research Unit, Greater Manchester Mental Health NHS Foundation Trust, Manchester, United Kingdom; ^3^Complex Trauma and Resilience Research Unit, Greater Manchester Mental Health NHS Foundation Trust, Manchester, United Kingdom

**Keywords:** voice hearing, Hearing Voices Network, peer support, NHS staff, survey

## Abstract

**Introduction:**

For over 40 years, Hearing Voices Groups (HVGs) have provided a space for individuals distressed by hearing voices to share their experiences openly. Most of these groups have existed in the community and adhere to a unique ethos which, at times, may be antithetical to that of mental health services. Recently, HVGs have started to be run within statutory services, including the UK’s National Health Service (NHS), raising questions about the practical and ideological barriers and facilitators to their successful implementation.

**Methods:**

NHS staff (*N* = 49) and HVG members (*N* = 26) took part in a mixed-methods survey aimed at understanding their perspectives on delivering HVGs in the NHS.

**Results:**

Overall, both staff and HVG members expressed enthusiasm for HVGs in the NHS, recognising their role in fostering peer connection, though staff raised concerns about risk management and HVG members questioned whether NHS-run groups could fully uphold HVG ethos.

**Discussion:**

Whilst HVGs offer a promising user-led approach, further research is needed to understand precisely how to run these types of groups in statutory services.

## 1 Introduction

Voice hearing, or the perception of speech/noise in the absence of an objective, external source (auditory hallucination), has long been considered a hallmark of psychiatric illness. Whilst voice hearing is most commonly associated with the diagnosis of psychosis or schizophrenia (e.g., American Psychiatric Association, 2013; World Health Organisation, 2019), it occurs transdiagnostically (de Leede-Smith and Barkus, 2013), and amongst those who have never come into contact with psychiatric services (Johns et al., 2002; Ohayon, 2000). Indeed, approximately 10% of the adult population report hearing voices at some point in their lives (Maijer et al., 2018), with only a subset only ever encountering psychiatric services or meeting diagnostic criteria for psychosis (Johns et al., 2014).

For over 40 years, Hearing Voices Groups (HVGs) have been providing distressed voice hearers with a source of support and community outside statutory mental health services (Dillon and Hornstein, 2013). The establishment of HVGs is one of the main prerogatives of the international Hearing Voices Movement (HVM), a sociopolitical movement of voice hearers and their allies which aims to shift mainstream conceptions of voice hearing away from a disease-based model and instead locates voice hearing as a meaningful and understandable human experience (Corstens et al., 2014). Historically, HVGs have positioned themselves as an alternative to traditional mental health services and accordingly, present a distinct ethos: for example, emphasising expertise by experience, validating subjective understandings of voice hearing (e.g., spiritual, technical, trauma-based, etc.) and learning to live peacefully alongside voices (Branitsky et al., 2020; Corstens et al., 2014; Dillon and Hornstein, 2013). In contrast, clinical services have historically positioned voice hearing as a marker of pathology and therefore prioritised voice cessation and a reduction in clinical symptomatology using medical interventions (e.g., medication; Sommer et al., 2012). Furthermore, unlike many clinical services, HVGs additionally place primary importance on establishing mutual relationships, do not offer pre-specified interventions, and are not outcome-oriented (Hornstein et al., 2020, 2024).

For most of their history HVGs have operated in the community, independent from clinical services (Corstens et al., 2014). Accordingly, there has been accumulating evidence that the benefits of these groups include increased social support (Corentin et al., 2023; Hornstein et al., 2024), hope for the future (Corentin et al., 2023; Hornstein et al., 2021) and an improved understanding of, and relationship to, both voices and oneself (Hornstein et al., 2024), all of which are valued and meaningful outcomes for voice hearers.

More recently, however, HVGs have also begun operating within statutory mental health services, such as the UK’s National Health Service (NHS; English Hearing Voices Network, 2024). Whilst the precise number operating in the NHS is unknown, there are groups currently running in both inpatient and outpatient services throughout the country. These groups tend to operate in an *ad hoc* nature, with individual services establishing groups where resources permit. The extent to which these groups adhere to the ethos of HVM is likewise unknown. For example, there is little documentation on whether these groups record clinical notes, if they are facilitated by professional or peer staff, and whether they utilise an unstructured format that allows for open discussion on topics beyond psychoeducation and coping. To date, only one reflective account describes the facilitation of an HVG across three inpatient units in the NHS. The group ran for 30–45 min per week, was facilitated by a peer support worker, and adhered to a structured approach which focused on sharing and learning coping skills (McManus et al., 2023). However, unlike their community counterparts, groups within statutory services have only recently begun to be studied. Existing results are promising, with the first investigation of online HVGs within statutory services in the US indicating that attendance reduced voice distress, improved beliefs about voices, and were seen as an important source of connection and hope (Kalofonos et al., 2023). The first feasibility study of an online HVG in the NHS similarly demonstrated the groups were feasible and acceptable, that group members particularly valued meeting other voice hearers and, crucially, that they offered a form of support that participants could not get elsewhere (Branitsky et al., 2025). A subset of participants emphasised the importance of having a peer-run space where they could share their experiences without the fear of consequences that can be present in clinical interactions (Branitsky et al., n.d.). Importantly, the online nature of the group did not act as in insurmountable barrier to connection or disclosure and indeed, some participants felt more comfortable with online participation because they could more easily modulate their participation by turning their camera off if desired and felt safer joining from the privacy of their home (Branitsky et al., 2024; Branitsky et al., n.d.).

Jones and Jacobsen (2022) conducted the first study of staff knowledge and attitudes toward HVGs in a sample of 40 multidisciplinary NHS mental healthcare workers. Most participants felt HVGs were valuable to both service users and staff; however, there was a discernible knowledge gap in professionals’ understanding of precisely what HVGs are, how they operate, and what the current evidence is for their utility. Whilst staff felt that HVGs reduced shame, improved hope, provided meaningful social contact and promoted overall recovery, some nevertheless expressed concerns about how HVGs managed risk and whether the ethos of HVGs were too divergent from those of the NHS (thus potentially leading attendees to start viewing statutory services as problematic). Similar findings were reported by Renaud et al. (2024), who found that whilst most mental health professionals endorsed positive views of HVGs, familiarity with HVGs was associated with a more positive view of the groups.

If HVGs were to be run more widely in NHS services, it is important to understand the potential ideological and practical barriers to successful implementation. The aim of this survey was therefore to understand both HVG members’ and NHS staff’s perspectives on: (1) perceived benefits of HVGs; (2) concerns about HVGs both in the community and the NHS; (3) the value and utility of online HVGs; and (4) the practical and ideological barriers to running HVGs in the NHS.

## 2 Materials and methods

### 2.1 Setting and participants

The study was approved by the NHS Surrey Borders Research Ethics Committee (24/PR/0140) with surveys conducted between April 5, 2024, and November 30, 2024. Two separate surveys were distributed: one for NHS staff and one for HVG members. Respondents were eligible to take part in the former if they were NHS staff who were employed as healthcare workers, regardless of discipline. For the HVG Member survey, eligible individuals: (1) were aged 18 or older; (2) self-reported current or past voice hearing; (3) currently attended a community-based HVG (either online or face-to-face) or had attended one in the past; (4) were able to provide informed consent; and (5) had sufficient comprehension of English to complete the survey.

The surveys were administered online via Qualtrics. Advertisements for the NHS Staff Survey were sent to service leads within the host Trust, presented at team meetings and posted on social media pages dedicated to NHS staff. Advertisements for the HVG member survey were sent to all group facilitators registered with the English Hearing Voices Network^1^ and were posted on social media [e.g., X (formerly Twitter)]. Both surveys took about 20 min to complete. Participants received written information about the study and indicated consent before proceeding. Due to the anonymous nature of the surveys, no compensation was offered.

### 2.2 Survey design and development

Data were collected through cross-sectional surveys focused on both NHS staff and HVG members’ views on running HVGs in the NHS. Surveys contained a combination of both open and closed questions. The survey structure and sample questions can be found in Table 1 for the NHS staff survey and Table 2 for the HVG member survey. The full NHS staff and HVG member surveys can be found in Supplementary materials 1, 2, respectively.

**Table 1:** NHS staff survey structure and sample questions.

Survey area	Scale type	Scale range	Sample question(s)
Familiarity with HVGs	Likert; open-ended	Strongly disagree – Strongly agree	“I have a good understanding of HVGs.” “I am aware of local HVGs (either online or face-to-face) that I can signpost service users to.”
Views of NHS versus community-run HVGs	Dichotomous; open-ended	Yes – No	“I would feel more comfortable referring service users to a HVG run within the NHS as opposed to a group run in the community.”
Perceived benefits of HVGs	Likert; open-ended	Not at all important – Very important	Please rate how important you think each item is: “Connecting with others with similar experiences.” “Learning new ways to engage with voices.” “Providing opportunities to speak about systemic oppression (e.g., racism, poverty, homophobia).” “Reducing stigma.”
Concerns around HVGs	Likert; open-ended	I am unconcerned – I am very concerned	Please indicate how concerned you are about the following: “Groups might encourage service users to stop taking their medication.” “Groups may reinforce individual’s delusional beliefs.” “I am unaware of how HVGs manage risk.”
Barriers and considerations for implementing HVGs in the NHS	Likert; open-ended	Strongly disagree – Strongly agree/ Much less likely – much more likely	Please indicate how much you think each of the following items are barriers to offering more HVGs within the NHS: “Lack of peer facilitators.” “Lack of resources to set up and facilitate online groups.” “Ideological differences between HVGs and mental health services.” Do you think any of the following features of groups would increase the likelihood of routinely implementing them into the NHS? “Having a structured, rather than unstructured, intervention.” “Integrated psychoeducation.”
HVG Facilitators	Trichotomous	Peers/ individuals with lived experience – Mental health professionals – Both	“Who do you think is best suited to facilitate HVGs?”
Perception of risk management in HVGs	Open-ended		“Do you have any thoughts about risk management in HVGs?”
Perceptions of online HVGs	Trichotomous; open-ended	Yes – No – Depends on the individual	“Would you be more inclined to refer somebody to an online HVG as opposed to a face-to-face HVG?”

**Table 2:** HVG member survey structure and sample questions.

Survey area	Scale type	Scale range	Sample question(s)
Reasons for attending and experiences in HVGs	Open-ended		“What made you want to attend your first hearing voices group?” “Please tell us about your experience in the hearing voices group.”
Interest in HVGs in the NHS	Dichotomous; open-ended	Yes – No	“Would you be interested in attending a hearing voices group that is run in the NHS?”
Important features of HVGs in the NHS	Likert; open-ended	Not at all important – Very important	If you were to attend a HVG in the NHS, how important are each of these features: “To meet other people with similar experiences.” “To be able to talk about difficult life experiences.” “For the group to be confidential.”
Concerns about HVGs in the NHS	Likert; open-ended	I am unconcerned – I am very concerned	If you were to attend a HVG in the NHS, would you be concerned about any of the following: “Talking about my voices might make them worse.” “My medication might get increased if I talk about certain things.”
Perceptions of online HVGs	Likert; open-ended	Strongly disagree – Strongly agree	In your opinion, some of the benefits of attending an online hearing voices group would be: “I feel safer/more comfortable at home.” “My voices feel safer/more comfortable at home.” “I would be able to have my camera off.” In your opinion, some of the challenges in attending an online HVG would be: “I’m concerned about others spying on me.” “I find it harder to connect with other people online.”

Both surveys were designed by the study team, which includes individuals with lived experience of voice hearing and HVG facilitation and participation. Additional input on items was also provided from Patient and Public Involvement and Engagement (PPIE) experts. Items were derived from the previous qualitative (Corentin et al., 2023; Hornstein et al., 2020, 2021, 2024), quantitative (Jones and Jacobsen, 2022; Longden et al., 2018) and theoretical (Corstens et al., 2014; Dillon and Hornstein, 2013) literature on HVGs; lived experience of voice hearing and HVG facilitation/participation within the study team; and PPIE consultation. All surveys were piloted with PPIE representatives prior to recruitment commencing; surveys were iterated following PPIE consultation to include: (1) questions about NHS staff members’ familiarity with HVGs; (2) questions specifically pertaining to perceptions of risk management; and (3) more free-text response options in both surveys.

### 2.3 Data analysis

As this survey took place online, effort was taken to mitigate the incidence of bot and fraudulent responses (Hitches et al., 2024). For the NHS staff survey, the survey was only posted to closed social media pages that were exclusive to NHS staff. Response rates were monitored following posting the survey to ascertain whether responses were likely to be genuine (e.g., an influx of responses in rapid succession may indicate fraudulent responses; Agans et al., 2023). For HVG member surveys, response rates were likewise monitored, with most responses submitted after emails to facilitators were sent and few submitted following social media posts. Descriptive statistics of quantitative data were generated to explore both staff and HVG members’ perspectives of HVGs in the NHS.

Free-text questions were analysed using deductive qualitative content analysis (Elo and Kyngäs, 2008). As the purpose of the qualitative analysis was to further contextualise and nuance the quantitative survey data, a manifest analysis was undertaken (Bengtsson, 2016) whereby the analysis and subsequent reporting adhered closely to the precise reports from study participants. Bengtsson’s (2016) approach to data analysis was followed: (1) decontextualisation whereby coding creates meaningful units in the data; (2) recontextualisation, where units are compared to textual data to ensure all content has been included; (3) categorisation where similar units are groups into categories and themes; and (4) compilation and reporting where findings can be presented from the data. Themes were created deductively based on relevant survey areas (e.g., benefits of HVGs in the NHS, concerns around HVGs in the NHS).

## 3 Results

### 3.1 NHS staff characteristics

A total of 64 NHS staff consented to take part, of whom 49 completed the survey. Demographic characteristics are presented in Table 3.

**Table 3:** NHS staff characteristics.

Characteristic	Total sample (*N* = 49)
Age (years) – mean (SD)	36.2 (10.4) [Range: 23–62]
Gender – *n* (%)	
Female	41 (83.7%)
Male	8 (16.3%)
Job role – *n* (%)	
Assistant psychologist	3 (6.1%)
CBT therapist	5 (10.2%)
Clinical psychologist	13 (26.5%)
Occupational therapist	2 (4.1%)
Peer support worker	1 (2.0%)
Psychiatrist	1 (2.0%)
Research assistant	1 (2.0%)
Senior nurse practitioner	3 (6.1%)
Support worker	1 (2.0%)
Trainee clinical psychologist	16 (32.7%)
Other mental health worker	2 (4.1%)
Not indicated	1 (2.0%)
Type of service – *n* (%)	
Child and adolescent mental health service	1 (2.0%)
Early intervention in psychosis	17 (34.7%)
Community mental health team	11 (22.4%)
Home-based treatment	2 (4.1%)
Inpatient	8 (16.3%)
National & specialist	1 (2.0%)
Research and innovation	4 (8.2%)
Other service	5 (10.2%)
HVG attendee on caseload – *n* (%)	
Yes	14 (28.6%)
No	30 (61.2%)
Unsure	5 (10.2%)

### 3.2 Staff survey results

Table format of presented data can be found in Supplementary materials 3.

#### 3.2.1 Familiarity and experience with HVGs

**Figure 1:**
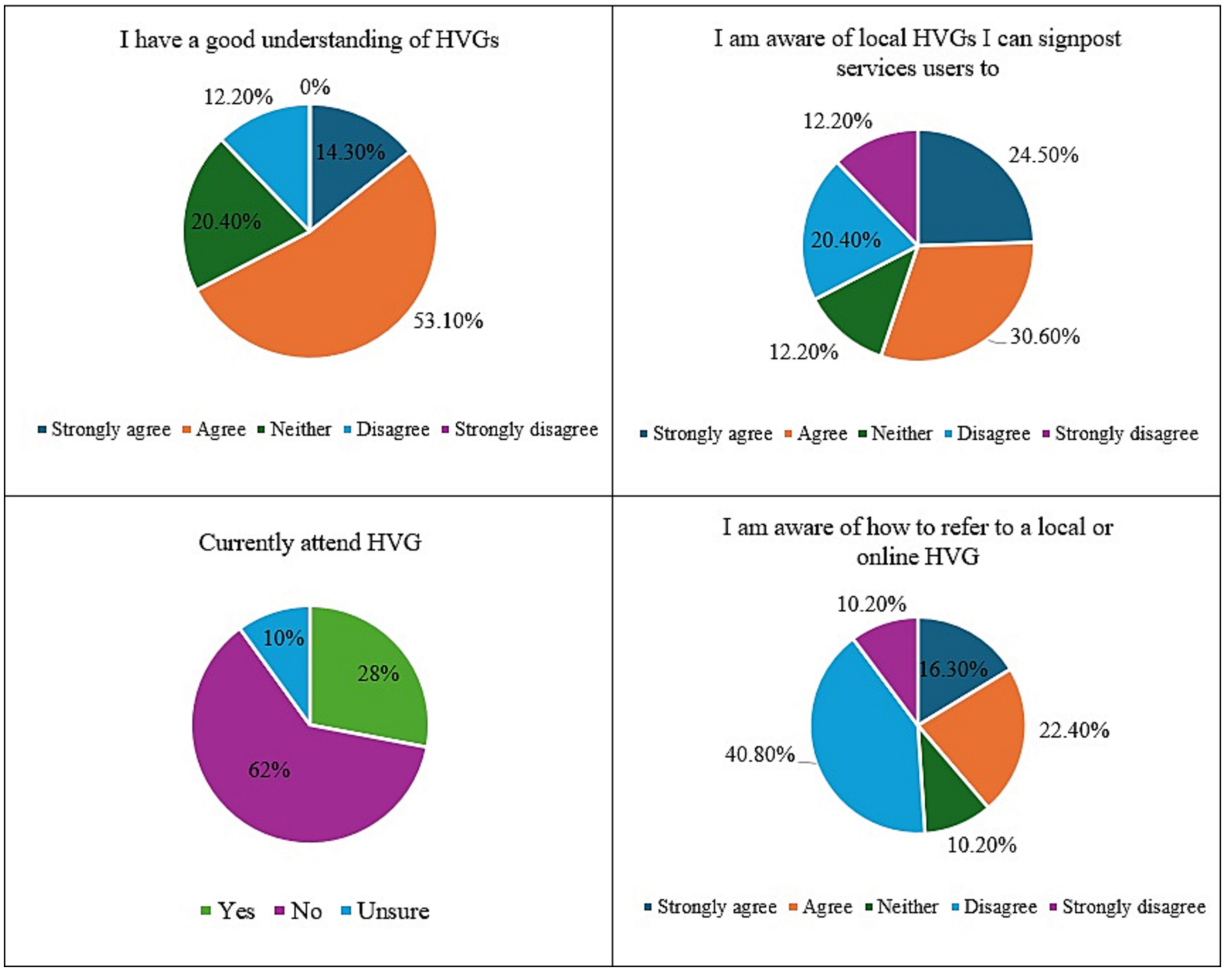
NHS staff familiarity with HVGs.

A summary of staff familiarity with HVGs can be found in Figure 1. Overall, most staff expressed familiarity with HVGs and reported that service users who attended them had positive experiences in the group. 67.4% of staff reported having a good understanding of HVGs, 55.1% were aware of local HVGs and 28% had service users on their caseload who were currently attending HVGs. However, most staff felt they were unaware of precisely how to connect interested service users with local HVGs.

Many staff stated that feedback they had received from service users about groups was “*extremely positive*” (P20). Connection was the most endorsed piece of feedback, with staff noting that groups offered “*a sense of community, safety, [and a] judgement free place to just be with other people who understand*” (P08). Through the “*social/peer aspect of the group*” (P10), staff reported that service users felt “*validated*” (P56), “*less alone*” (P42) and that the group afforded them “*a safe space to talk and reflect [and] discuss the impact of experiences and coping strategies*” (P52). Notably, staff did not remark on the groups providing an alternative form of support, except for one participant who noted that HVGs may be particularly beneficial because they provide “*a different perspective that’s more hopefu*l” (P22). Whilst staff mostly reported positive impressions, one respondent did note that feedback from service users had been “*mix[ed]*” wherein “*some enjoyed speaking about their experiences and finding like-minded people, some have felt the other individuals felt quite different to themselves*” (P02).

#### 3.2.2 Perceived benefits of HVGs

**Figure 2:**
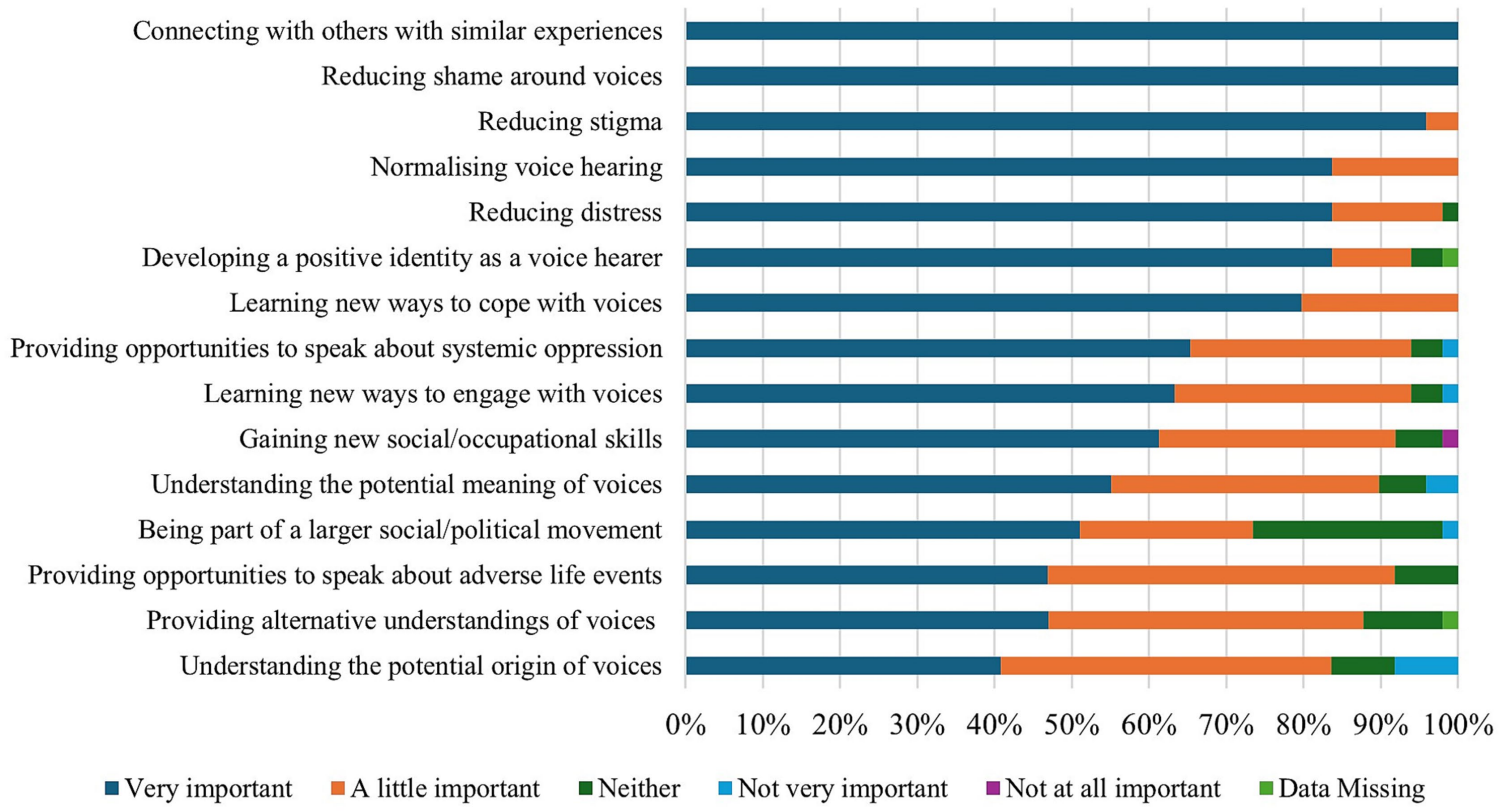
NHS staff perspectives on the relative importance of HVG benefits.

Staff likewise endorsed many benefits of HVG attendance (see Figure 2), including meeting other voice hearers, reducing shame and stigma around voice hearing, normalising voice hearing, and reducing distress. Whilst staff felt the most important benefit of HVGs was connecting with other voice hearers, elements central to the ethos of HVGs such as exploring the potential meaning, origin, and life events associated with voice hearing, were rated as slightly less important.

Staff also noted other pragmatic benefits to attending HVGs, such as “*routine*” (P53), “*connecting with others socially*” (P42) and the group simply being “*something to do*” (P43). On a more emotional level, staff additionally stated that, “*being able to feel compassion for others in a similar position to yourself can foster self-compassion where just speaking with a therapist sometimes does not*” (P56). Furthermore, they noted the significance of “*hearing others use words or questions to describe experiences/emotions that you are finding hard to express*” (P29) and feeling like you can usefully “*contribute*” (P53) to others.

#### 3.2.3 Community versus NHS HVGs

Despite these positive views, 38% of respondents reported feeling more comfortable signposting service users to HVGs run in the NHS as opposed to in the community. Most of these concerns related to perceptions of training and quality control, with one respondent noting “*NHS groups would have had training…whilst community groups tend to be inconsistent and* var*y in content and might not have the same level of training*” (P03) and another reporting “*I feel the NHS would be more reliable in terms of the quality of intervention they are providing*” (P59). Some expressed concerns that attending community-based HVGs carried risks:

*I worry about HVGs outside the NHS being more likely […] to adopt an anti-service ethos, potentially encouraging a few people into disengagement, non-adherence with medication and even admission now and again.* (P21).

However, others noted that “*being by the NHS is not necessarily a guarantee of quality control*” (P35) and there may be unique benefits of individuals attending community-groups: “*groups being independent [may enable] people feeling more free to speak,* e.g.*, without fear that information will appear on their notes and then the psychiatrist would want to increase their medication*” (P42). Furthermore, some staff members felt the ethos of HVM was more easily applied in HVGs that operated outside the NHS: “*I think HVGs feel more suited to a community approach than within the structure of the NHS, I’d worry about the power balance of clinicians vs [experts by experience]* (e.g.*, too much clinician/risk focus to allow space for open and honest discussion*)” (P58).

Most participants stated there were “*pros and cons*” (P23) of each type of group but emphasised that to signpost to community-based groups, the group would need to be “*well-organised and facilitated by experienced/well reputed group facilitators*” (P35). Staff likewise highlighted that service users’ “*needs and wishes*” (P23) would impact whether they would signpost to a community group, with one respondent noting:

*People should be given every opportunity and repeated opportunities to engage with such a group. It may not always be the right time for people when they are acutely distressed in hospital and may not be able to cope with others’ distress/sad stories but it should be an offer. For others, it may be the perfect time and prevent secondary problems associated with the meaning attached to psychosis, such as social exclusion, loss of self-esteem, hopelessness*. (P56).

**Figure 3:**
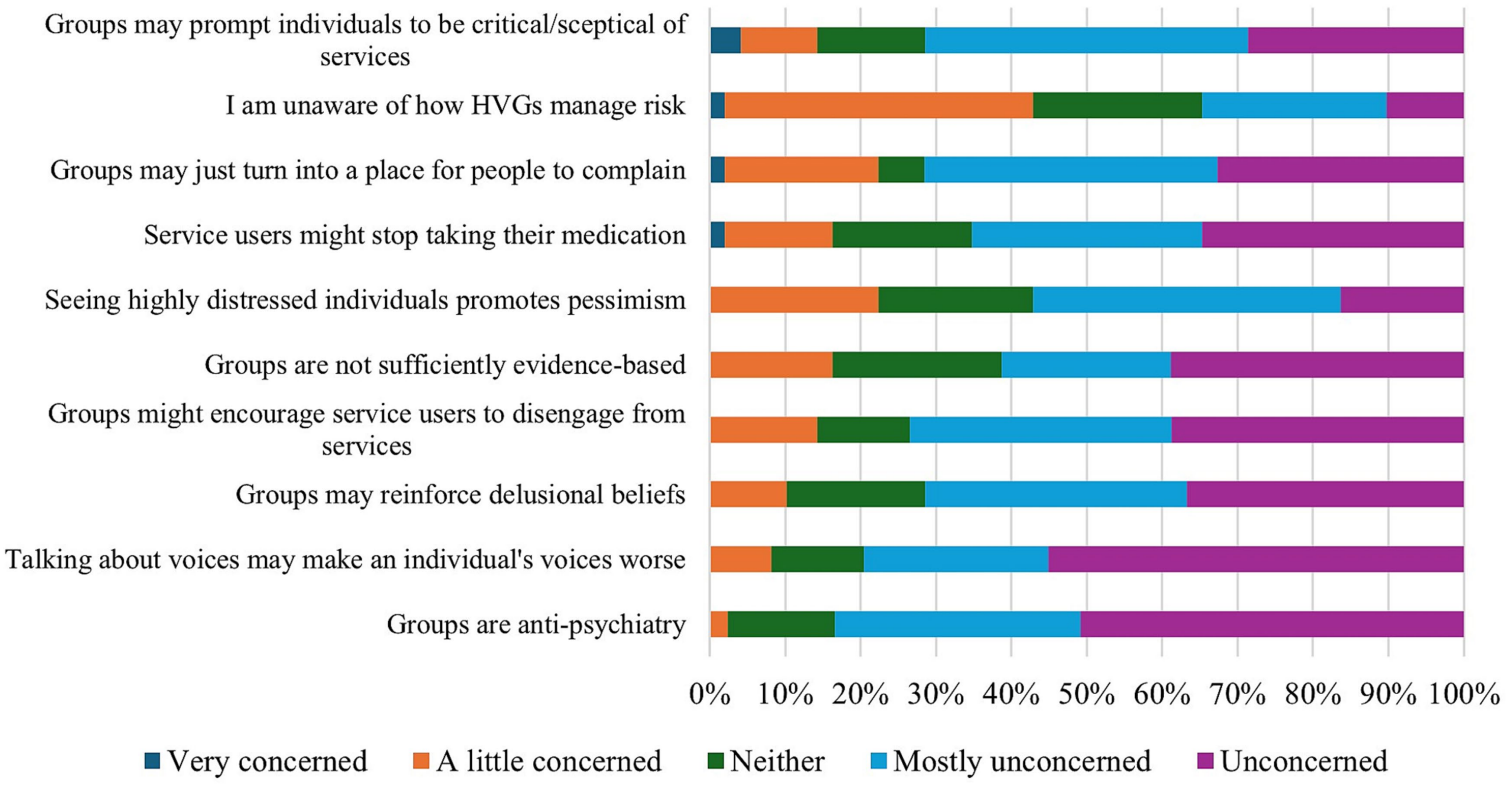
NHS staff concerns about HVGs.

Staff generally endorsed few concerns about HVGs. The most strongly endorsed concern regarded a lack of awareness of how HVGs manage risk (see Figure 3), and many elaborated that they would feel more comfortable signposting voice hearers to a community group if they could be assured that the facilitators were able to respond appropriately to distress.

Respondents requested reassurance surrounding “*group regulations regarding management of risk/person in crisis*” (P27) and some suggested groups “*cover risk management of self and others in agreed group ‘ground rules’*” (P10) and that “*it is good if everyone is offered a safety plan*” (P09). Some participants likewise noted that their perspectives around risk depended on “*whether [the HVG] as within or outside the NHS*” (P23) with groups held within the NHS requiring “*local policies*” (P08) to align to the NHS “*duty of care*” (P54) to service users, whilst in community groups “*signposting and common sense should suffice*” (P43). However, for most staff (96%), however, these concerns would not prevent them from referring a voice hearer to a community-run HVG.

Staff likewise expressed few reservations about signposting voice hearers to online HVGs; 81% of staff reported that referral decisions for an online group rather than a face-to-face group would be based on voice hearer preference. Staff noted that would seek to ensure “*someone’s online security and confidentiality was protected*” (P23) but reported no further concerns that were unique to the online medium.

Finally, whilst many staff endorsed positive views of HVGs, not all staff believed that they should be run in the NHS. Staff highlighted the distinct ethos, noting that it would likely be compromised in NHS settings. One participant noted: “*I suspect people need their space away from us to be able to speak freely. If that was eroded where would they go?*” (P42).

#### 3.2.4 Barriers to HVG implementation in the NHS

**Figure 4:**
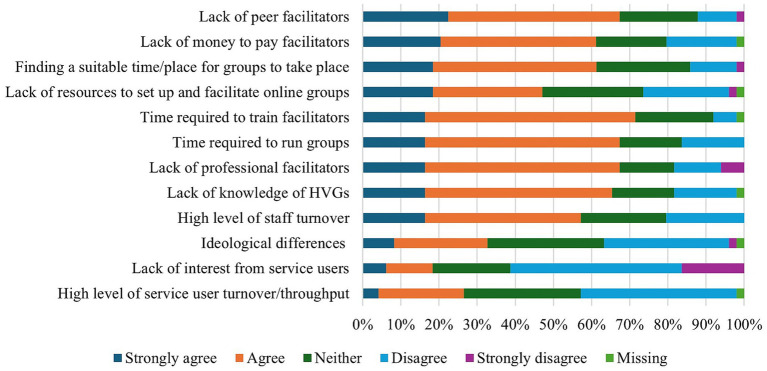
NHS staff perceptions of the barriers to HVG implementation in the NHS.

As presented in Figure 4, resource limitations were commonly cited as barriers to HVG implementation in the NHS. Specifically, 97% of staff felt that HVGs should be facilitated or co-facilitated by a voice hearer yet noted that a lack of peer facilitators was a significant challenge. Staff further suggested that NHS trusts need to compensate peer facilitators. For example, one staff member suggested trusts *“[make] the expert by experience facilitator a paid role rather than voluntary*” (P23). Ideological differences between HVGs and the NHS was generally not seen to be a barrier, although one participant did note that “*perceptions that it is not fitting with the medical model*” (P24) may pose a challenge.

Additional barriers to NHS implementation noted by staff included “*governance challenges re using experts by experience*” (P22), “*a risk-averse attitude [that] prevents innovation and change*” (P61), the fact that “*mental health services [are] ‘fire-fighting,’ without sufficient time/resource/reflective thinking space to consider referring people to groups*” (P29) and the need for “*more evidence that patients want/like them and they are beneficial*” (P45). One member of staff felt the most compelling case for groups being implemented into the NHS was establishing stronger “*evidence in favour of peer support within clinical settings*” (P61).

**Figure 5:**
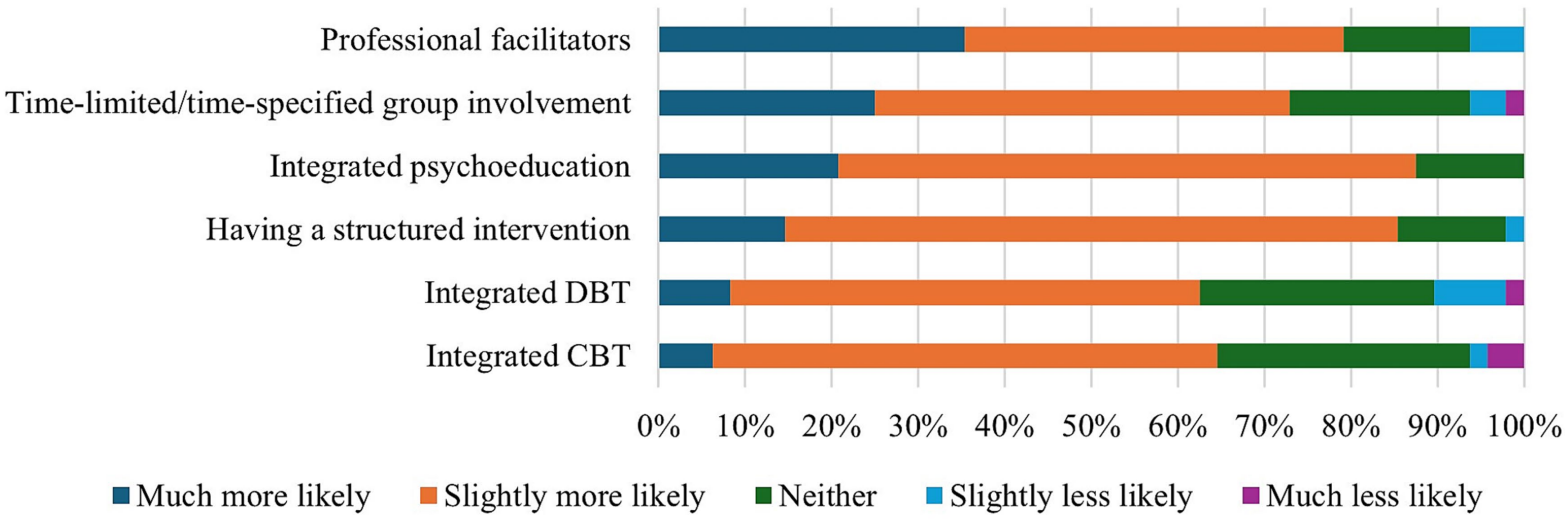
NHS staff perceptions on the factors that would increase the likelihood of HVG implementation.

Finally, staff felt that structuring groups to fit more closely with existing NHS provision may help with implementation (see Figure 5). However, whilst it was noted that some of these features would increase the likelihood of HVGs being run in the NHS, not all staff felt it was wise to adapt groups to such an extent:

*I have rated time-limited and structures as increasing likelihood [of implementation] but I do not think they are a good idea. Integrating CBT and DBT would be much more resource-intensive and also a bad idea [in my opinion]. I believe people need a forum away from structured intervention.* (P43).

### 3.3 HVG member characteristics

A total of 35 HVG members consented to take part, of whom 26 completed the survey. Demographic characteristics are presented in Table 4.

**Table 4:** HVG member characteristics.

Characteristic	Total sample (*N* = 26)
Age (years) – mean (SD)	46.3 (12.4) [Range: 22–66]
Gender – *n* (%)	
Female	19 (73.1%)
Male	6 (23.1%)
Non-binary	1 (3.8%)
Ethnicity – *n* (%)	
Black	2 (7.7%)
Asian	1 (3.8%)
White Caucasian	21 (80.1%)
Mixed-Race	2 (7.7%)
Age (years) voice hearing started – mean (SD)	21.8 (14.4) [Range: 0–42]
Duration of voice hearing (years) – mean (SD)	25.8 (20.0) [Range: 2–58]
Number of voices – *n* (%)	
One	3 (11.5%)
Two-five	13 (50.0%)
Six-ten	3 (11.5%)
More than 10	5 (19.2%)
Not indicated	2 (7.7%)
Self-reported diagnoses – *n* (%) (participants could endorse multiple diagnoses)	
Schizophrenia	10 (38.5%)
Schizoaffective	3 (11.5%)
Psychosis	6 (23.1%)
Dissociative identity disorder	1 (3.8%)
Bipolar disorder	8 (30.8%)
Borderline personality disorder	5 (19.2%)
Depression	8 (30.8%)
Anxiety disorder	6 (23.1%)
Complex post-traumatic stress disorder	5 (19.2%)
Post-traumatic stress disorder	1 (3.8%)
Obsessive compulsive disorder	1 (3.8%)
Dermatillomania	1 (3.8%)
Eating disorder	1 (3.8%)
Current involvement with NHS mental health team – *n* (%)	
Community mental health team	12 (46.2%)
Home based treatment team	1 (3.8%)
None	11 (42.3%)
Not indicated	2 (7.7%)
Type of HVG attended – *n* (%)	
Community	8 (30.8%)
NHS	0 (0%)
Online	16 (61.5%)
Unsure	2 (7.7%)

### 3.4 HVG member survey results

Table format of presented data can be found in Supplementary materials 4.

#### 3.4.1 Motivations for attendance

Participants reported multiple reasons for attending their HVG, although the majority cited seeking some form of shared experience as the most important reason, with peer support being identified as uniquely beneficial. One person simply claimed, “*I wanted to talk to other people and hear their experiences*,” (P10) whilst others believed that meeting other voice hearers would reduce their own sense of isolation, “*to hear other’s experiences and to know I wasn’t alone*” (P03). Peers with shared experiences were perceived as more likely to understand other’s experiences; for example, one participant stated: “[I] *need support and wanted to talk about my experiences with people who would likely understand*” (P19) whereas another seconded, “*being able to hear from other people who are affected by [voice hearing] as talking to people who have never experienced it is difficult*” (P12). Other motivations for attendance included desire to foster community (“*I wanted to attend to find community and solidarity, mutual understanding and support*” [P21]), reduce distress (“*another source of support and understanding for the distress I was experiencing with the voices*” [P05]), and contribute to the wellbeing of others (“*[I have a] desire to volunteer as I teach meditation and thought I could share some anxiety hacks*” [P02]).

#### 3.4.2 Perspectives on HVGs in the NHS

**Figure 6:**
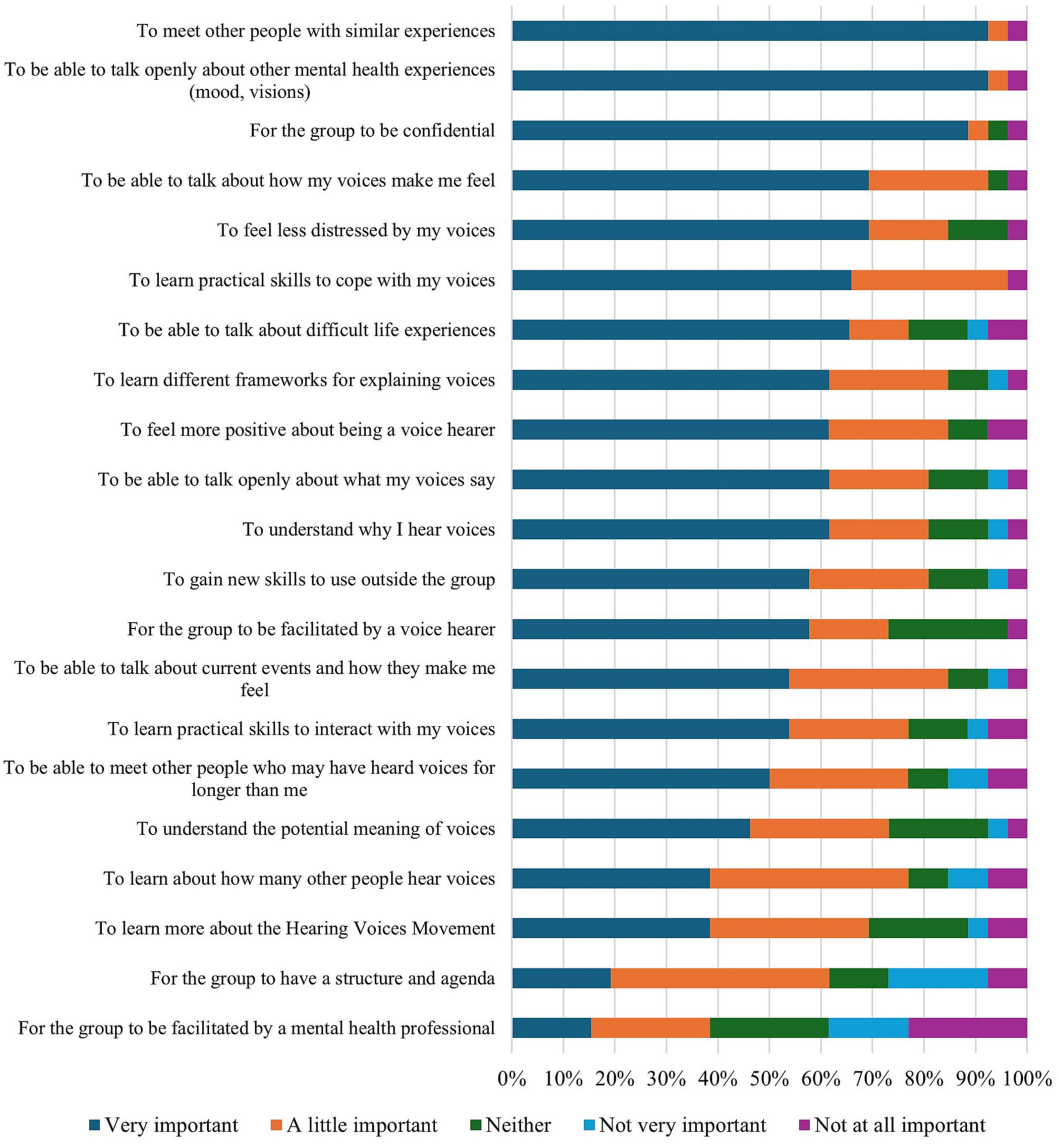
HVG participants’ endorsement of important features of HVGs in the NHS.

Figure 6 presents features that participants rated as important for HVGs operating within the NHS. In this respect, the majority (69.2%) expressed an interest in attending such a group; however, over a quarter (26.9%) were not interested in attending a group run in the NHS and 2.8% were unsure. Of those who were interested, several participants felt that holding groups within clinical services would “*increase accessibility*” (P05) and enable more voice hearers to “*gain access*” (P02). On a practical level, participants believed that NHS groups would “*have more people which would make the atmosphere and support received better*” (P11) and that these groups would decrease the burden of having to find a community group, especially if someone was acutely distressed: “*when you are in your illness badly, an NHS one could be a good first step to aid recovery*” (P06). One participant hoped that having HVGs in NHS would serve to further legitimise the HVM approach: “*The NHS could spread the word very effectively and give HVGs a stamp of approval that might reassure that it’s a safe place*” (P02).

**Figure 7:**
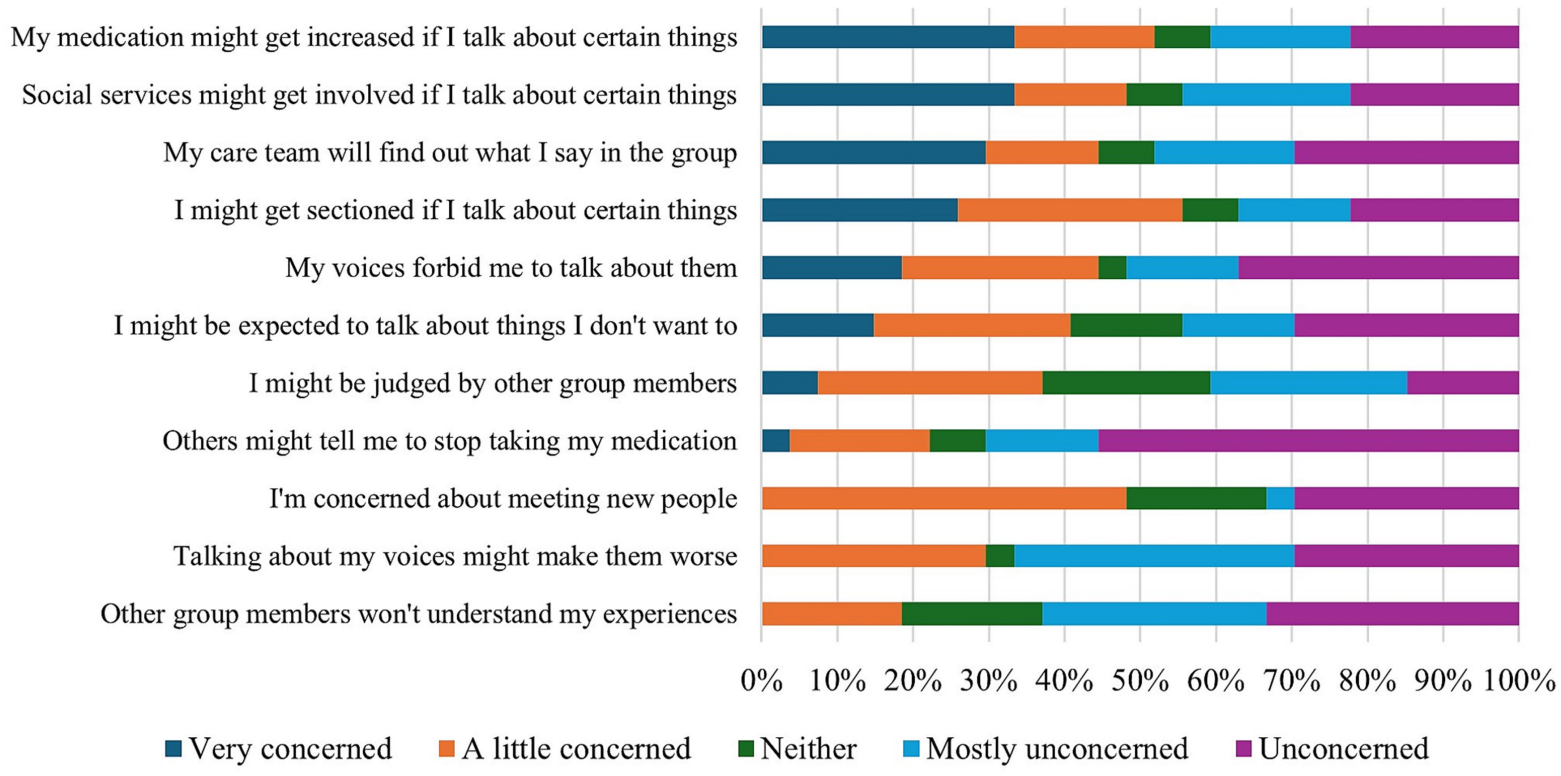
HVG participants’ concerns about attending HVGs in the NHS.

However, other participants expressed significant concerns about HVGs being run in the NHS, primarily from the potentially harmful consequences, such as increased medication, that could result from a lack of true confidentiality (see Figure 7). In their written responses, participants further noted that the “*ethic of control*” (P14) which manifests in some clinical services is antithetical to that of HVGs and may thus render it “*impossible*” (P22) to run such groups in NHS services. Voice hearers expressed concern that “*with the NHS […] when setting up a HVG they are constrained by their organisation first rather than the voice hearers’ needs*” (P22). They therefore questioned whether peer values could be authentically implemented in clinical services:

*I think being led by NHS staff who have no first-hand experience of voice-hearing limits the sense of freedom everybody feels to speak openly and honestly […] the staff in the NHS group used to make notes after each session for ‘risk management,’ and this made some people uncomfortable to share their true feelings and experiences.* (P21).

Even those who supported HVGs in the NHS noted that they would need to be “*clearly differentiated from Hearing Voices Network groups because the ethos is different*” (P22). Numerous participants expressed concerns about the “*lack of honest confidentiality*” (P15) in groups which are not purely peer led, and that the diagnostic emphasis within clinical services is antithetical to the open exploration of experiences promoted by the HVM. As one participant noted:

*Some facilitators [are] too keen to bring therapeutic techniques into the session. NHS groups do not encourage attendees to work out their voices by themselves, to learn from the voices. Instead attendees tend to place a reliance on the NHS facilitator. I think a similar situation would evolve if the facilitators were peers, just the NHS environment would not encourage the free flow of discussion and ideas and support of true peer led groups.* (P15).

Participants emphasised that if groups are run in the NHS, facilitators should be trained by voice hearers, group should ideally be peer led, and at a minimum, have a continuity of facilitators week-to-week. Furthermore, it was felt that groups should not be exclusively premised on the medical model but rather “*acknowledge that there may be* var*ious reasons for voices to exist*” (P15). Participants likewise highlighted the importance of maintaining the “*social*” (P06) element of the group, such as promoting “*friend[ships]*” (P07) beyond it, and being mindful that preventing members from attending the group post-discharge could represent a “*gut wrenching*” (P21) loss of community.

#### 3.4.3 Perspectives on online HVGs

**Figure 8:**
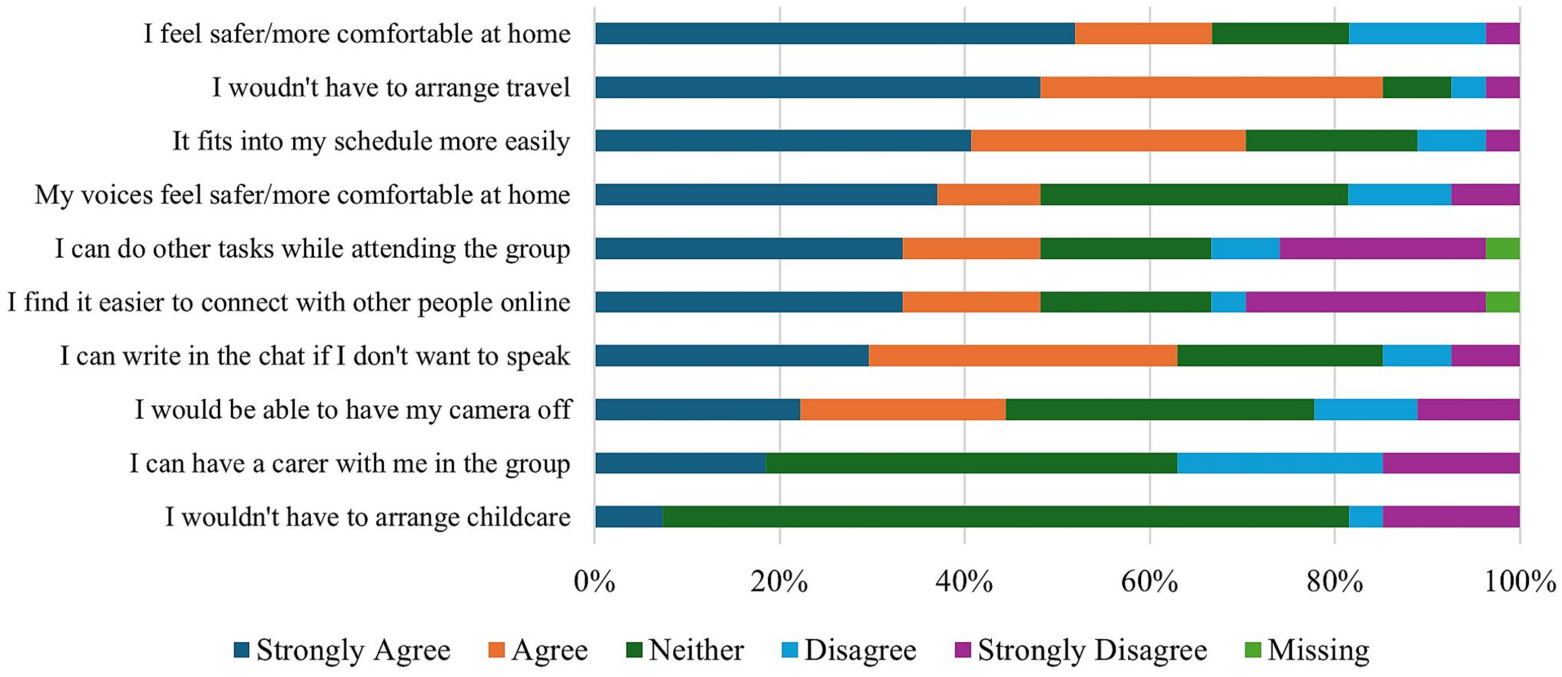
HVG participants’ perceptions of the benefits of online HVGs in the NHS.

Participants did not express a clear preference with regards to the form of group meetings, with 10/26 (38.5%) favouring online HVGs, 9/26 (34.6%) face-to-face groups, and 7/27 (26.9%) having no preference. Of those who preferred online attendance, increased accessibility and comfort were cited as the primary benefit. For some participants, physical disability made attending face-to-face HVGs more difficult: “*I have a physical disability and am often unable to schlep out to physical groups – I am more likely not to go to a physical group last minute for the reason of money/time and physical energy expense*” (P08). Attending from home likewise helped participants “*feel safer and more comfy*” (P23) and lowered the bar for participation because members “*can leave whenever [they] want and do not feel pressurized by others to stay*” (P10). The perceived benefits of online HVGs are reported in Figure 8.

**Figure 9:**
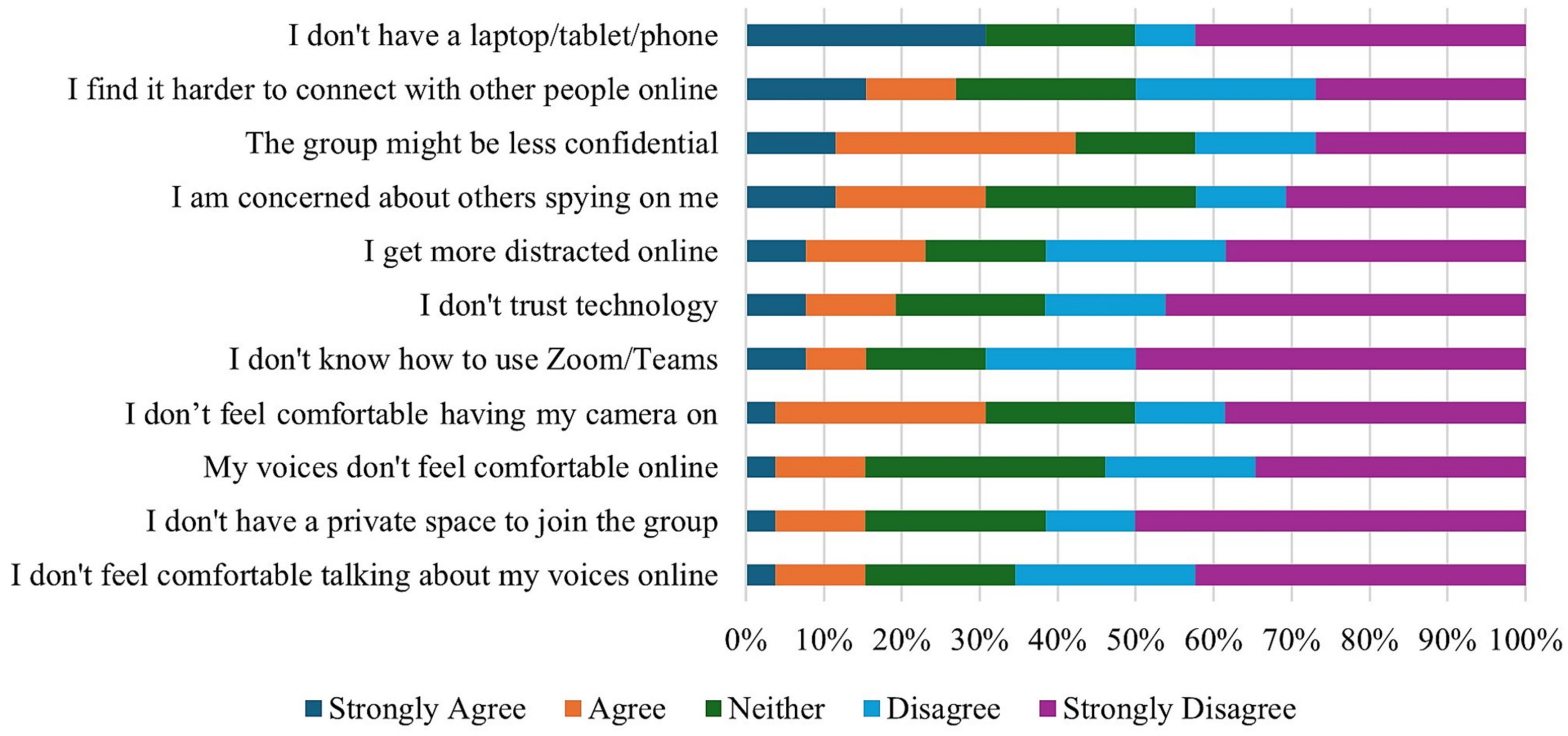
HVG participants’ perceptions of the challenges with online HVGs.

Despite the benefits of online groups, some participants nevertheless expressed concerns with the medium (see Figure 9). For example, beyond the “*occasional technical problem*” (P20), participants noted challenges with connecting to others online, with some *“[finding] it harder to interact with people online and tend[ing] to shut down and say less*” (P12) whilst others reported getting “*easily distracted*” (P16). Furthermore, the online medium could likewise have detrimental interactions with distressing mental health experiences, with one participant noting that online groups would bring up their “*fear of being spied on, using my camera [and] not trusting others*” (P21). Other members reported “*increased paranoia*” (P21) and “*voices [that] do not like me doing anything online*” (P25), though it was noted that these distressing experiences may be “*overcome*” (P15) by repeated attendance.

## 4 Discussion

This study is the first to investigate both staff and voice hearer views on HVGs run within nationally funded mental health services. Overall, staff endorsed positive views and expressed enthusiasm about groups being run more widely within the NHS specifically. They did, however, report concerns about risk management and noted that the lack of peer facilitators, and lack of funding to pay them, would likely be barriers to implementation. HVG members were likewise open to attending groups run within the NHS, with 69.2% saying they would consider attending an NHS-run group.

Both staff and HVG members cited connecting with other voice hearers as the most important feature of HVGs, which is consistent with previous literature that highlights their utility in providing a place to connect with others with similar experiences (Branitsky et al., 2020; Corentin et al., 2023; Hornstein et al., 2020, 2024). In contrast, no HVG member cited learning new skills as their primary reason for attending. Both the opportunity to learn new skills and the structured nature of the group were considered less important than the chance to connect with others. By extension, it is possible that this desire for connection over skills is an indirect indication for a preference for a non-directive and democratic group structure. This finding is noteworthy, as most staff respondents indicated that having a structured intervention, which incorporates psychoeducation, CBT, or DBT into the groups, could potentially enhance the likelihood of HVGs being more widely implemented. This discrepancy indicates that when NHS staff are planning to run HVGs, they should be advised that an unstructured approach is not only preferable but also aligns with the HVM ethos (Dillon and Hornstein, 2013). Consequently, the primary emphasis should be on fostering connections between group participants.

Similarly, although 69.2% of HVG attendees expressed interest in attending NHS-run groups, their primary motivation was practical and pragmatic reasons, rather than a belief that these groups would provide different or superior benefits compared to community-run groups. However, staff generally did not report well-resourced or well-attended groups within the NHS. This suggests that, at this time, the NHS may not be ideally positioned to offer groups that are both well-resourced and aligned with the HVM ethos.

Perspectives on clinical risk were also a notable point of difference between NHS staff and HVG members. Consistent with previous research on healthcare workers’ attitudes toward HVGs (Jones and Jacobsen, 2022), NHS staff noted that their greatest concern with either referring individuals to HVGs or to running them in the NHS focused on risk management and feeling unsure about how HVGs managed risk. However, the most commonly endorsed concern for HVG members was the reporting of group content to members of their care team, which they feared would result in the involuntary increase in medication, hospitalisation, or the involvement of social services, whilst noting that if groups were to be run in the NHS, then establishing strict confidentiality was paramount. As such, confidentiality and risk management would need to be carefully considered if groups were to be run more widely in the NHS. Adopting the HVM-aligned Alternative to Suicide approach (Davidow and Mazel-Carlton, 2020), a peer support approach in which individuals are given space to speak openly about thoughts of suicide and explore its potential meaning, may warrant further consideration.

In a related point, many staff who said they would be more comfortable referring services users to NHS-run HVGs rather than community-based ones also cited confidence in the facilitators’ training as a key reason for their preference. Whilst staff did not indicate precisely what they thought sufficient training would entail, their expressed concerns make it reasonable to infer that such training would prioritise risk and distress management. Conversely, however, HVG members reported reservations around NHS facilitators being too risk-averse and emphasised that if groups were to be run in the NHS, facilitators should receive training that focuses on the values and ethos of HVGs. Given that few NHS staff have attended HVG facilitator trainings (English Hearing Voices Network, personal communication, 2024), this may be an important avenue for exploration for interested staff members.

As online forms of support grow more prevalent, it is important to consider HVG members and NHS staff’s attitudes toward online HVGs. Amongst HVG members, preferences for connecting varied. Nearly half of the respondents reported finding it easier to connect online, whilst nearly a third found it more challenging. Online groups were perceived to yield many additional benefits, including reducing the travel burden and promoting feelings of safety for both voices and voice hearers. Previous research (e.g., Beck et al., 2023; Branitsky et al., 2024) on online support groups broadly, and HVGs specifically, highlights that the choice around participation afforded by the online medium (e.g., choice around camera use) enables participants to modulate their engagement, which may be particularly appealing to those who are hesitant to attend. Whilst previous research conducted with HVG facilitators indicates that technology-based distressing beliefs were not as significant a barrier to online participation as anticipated, the results from the current survey indicate that, whilst potentially uncommon, some HVG attendees do indeed endorse these fears and choice around preferred medium is therefore necessary to ensure that all participants can engage in a way that is suitable to them and their needs. Similarly, NHS staff did not express any concerns with online groups beyond ensuring confidentiality and appropriate risk management, with the majority claiming that they would be willing to refer a service user to an online HVG if that was their preference. Considering that online HVGs may offer a more resource-efficient form of support that addresses both the practical and emotional needs of voice hearers, fostering the development of these groups could be a valuable way to promote individual choice.

Nevertheless, when considering running HVGs in the NHS, it remains important to be mindful of the ideological differences in their approaches to voice hearing. Indeed, HVG attendees expressed concerns that the NHS would not be able to uphold the ethos of HVGs because of its hierarchical structure and regulatory requirements. Some staff echoed this sentiment, believing that the groups were better suited to community-settings where their peer values would not be compromised by NHS policies. Where HVGs are already being run in the NHS, these groups should ensure they place central importance on explicitly discussing the ethos of HVGs, fostering mutual relationships between group members, allow space for members to speak openly about all mental health experiences in addition to voices, as well as difficult life experiences, and be open and curious about subjective explanations for voice hearing. NHS-run groups should likewise be clear about the limits of confidentiality and policies around risk management and make efforts to refer interested service users to groups that can more closely adhere to HVM ethos.

### 4.1 Limitations

There were several limitations. The sample size was small and therefore may not represent the full diversity of perspectives of either HVG members or NHS staff members. Staff respondents were primarily psychological practitioners who were familiar with HVGs, which may have resulted in an over-representation of positive endorsements of HVGs compared to the larger NHS workforce. Therefore, the generalizability of the study results is severely limited. Study findings would have been strengthened by recruiting a more diverse group of professionals, particularly medical professionals, who arguably represent the majority of healthcare providers that voice hearers may have encountered in services. As these professions tend to be more rooted in a biomedical discourse, capturing their perspectives on HVGs would likely have reflected a more accurate representation of service-level opinions toward HVGs. In turn, there are also several domains that warrant more detailed consideration; for example, whilst most staff felt that incorporating psychoeducation would make groups easier to implement, it is unclear what specific type of psychoeducation they believed would be favourable. HVG members reported experiencing voice hearing for an average of 25.8 years. This long-standing experience likely influenced their reasons for seeking out HVGs and shaped their perspectives on HVGs within the NHS. Individuals with prolonged voice hearing tend to prioritise connecting with others over learning practical coping strategies (Branitsky et al., n.d.; Morrison et al., 2023). Furthermore, 80.1% of HVG member respondents identified as White Caucasian, which may not be reflective of the general population of voice hearers in the UK, or those who attend community-based HVGs. Given that those from ethnically minoritized groups are more likely to be given a psychosis diagnosis (Public Health England, 2016), the findings would have undoubtedly been strengthened by recruiting a more ethnically diverse sample of HVG members. Finally, whilst efforts were made to recruit a sample of NHS service users to garner their perspectives on HVGs in the NHS, only two responses were collected and therefore were excluded from the analysis. This precluded a comparison in preferences between HVG members and NHS service users who hear voices but have not had contact with community-based HVGs, which may have elicited important information about how to combine and complement the perspectives of both groups.

## 5 Conclusion

Overall, both staff and voice hearers expressed positive views and enthusiasm toward HVGs in the NHS, with groups specifically praised for being a space for voice hearers to connect with others with similar experiences. However, staff members did raise concerns around risk management in HVGs, whereas HVG members queried whether NHS groups could remain truly confidential and adhere to the values of the HVM. Ongoing research with representative samples is needed to consider strategies to resolve the tension between risk and recovery-oriented approaches to voice hearing, thus ensuring that if HVGs continue to be implemented within NHS services, that their ethos is not so compromised as to erode the benefits of these types of groups.

## Data Availability

The raw data supporting the conclusions of this article will be made available by the authors, without undue reservation.

## Footnotes

^1^ https://www.hearing-voices.org/hearing-voices-groups/find-a-group
