# Supplementary material for: Staff and voice hearer perspectives on Hearing Voices Groups in the NHS: a mixed-methods cross-sectional survey
Source: Front Psychol. 2025 Jul 4;16:1583370. doi: 10.3389/fpsyg.2025.1583370 (PMC12271199; doi:10.3389/fpsyg.2025.1583370)
Supplement: Supplementary file 1 [file Table_1.docx]

Supplementary Material 1

# Supplementary Material 1. NHS Staff Survey

**Hearing Voices Groups in the NHS – Staff Perspectives**

Thank you for taking the time to complete this survey. Your answers to this survey are anonymous. Please do not include any personal identifiable information in the free text boxes.

Hearing Voices Groups (HVGs) have been running in the community for over 30 years. These groups are generally peer-led, unstructured groups where individuals can speak openly to others about their voice hearing experiences. Groups do not follow any type of treatment plan, members can come for as often as suits them, and there are no prespecified outcomes that groups aim to achieve. We are curious to see if any how these types of groups may be implemented more widely into the NHS.

1. Age _________________
2. Gender ___________________
3. Trust ________________
4. Job role _________________
5. Which service do you work in:

| - EDIT |  | - Early Intervention |  | - CMHT |  |
| --- | --- | --- | --- | --- | --- |
| - Inpatient Services |  | - Residential Care |  | - Other (Please describe) |  |

1. Do you have individuals on your caseload who currently attend HVGs?

| - Yes |  | - No |  | - Unsure |  |
| --- | --- | --- | --- | --- | --- |

1. Service users on my caseload currently attend (please check all that apply):

| - Hearing Voices Groups | |  |
| --- | --- | --- |
| - 12-Step group (e.g., Alcoholics Anonymous) | |  |
| - CBT groups | |  |
| - DBT groups | |  |
| - Perinatal groups | |  |
| - Trauma survivor groups | |  |
| - Social/occupational skills groups | |  |
| - Identity-based support groups (e.g., LGBTQIA+ groups) | |  |
| - Other peer support group | |  |
| - Other (please indicate): |  | |

1. Have service users ever shared feedback about HVGs they’ve attended? If so, what was your impression of the group?

|  |
| --- |

Please indicate how much you agree with each of the following:

|  | - Strongly Disagree | - Disagree | - Neither Agree nor Disagree | - Agree | - Strongly Agree |
| --- | --- | --- | --- | --- | --- |
| 1. I have a good understanding of HVGs |  |  |  |  |  |
| 1. I am aware of local HVGs (either online or face-to-face) that I can signpost service users to |  |  |  |  |  |
| 1. I am aware of how to refer to a local or online HVG. |  |  |  |  |  |

1. I would be more comfortable referring service users to a HVG run within the NHS as opposed to a group run in the community.

| - Yes |  | - No |  |
| --- | --- | --- | --- |

Please tell us more about this response.

|  |
| --- |

Below are some potential advantages of attending HVGs. Please rate how important you think each item is:

|  | - Not at all important | - Not very important | Neither important nor unimportant | - A little important | Very important |
| --- | --- | --- | --- | --- | --- |
| 1. Connecting with others with similar experiences |  |  |  |  |  |
| 1. Reducing shame around voices |  |  |  |  |  |
| 1. Reducing stigma |  |  |  |  |  |
| 1. Reducing distress |  |  |  |  |  |
| 1. Normalising voice hearing |  |  |  |  |  |
| 1. Understanding the potential meaning of voices |  |  |  |  |  |
| 1. Understanding the potential origin of voices |  |  |  |  |  |
| 1. Learning new ways to cope with voices |  |  |  |  |  |
| 1. Learning new ways to engage with voices |  |  |  |  |  |
| 1. Providing alternative understandings of voices beyond those of mental health services (e.g., spiritual, cultural) |  |  |  |  |  |
| 1. Providing opportunities to speak about adverse life events |  |  |  |  |  |
| 1. Providing opportunities to speak about systemic oppression (e.g., racism, poverty, homophobia) |  |  |  |  |  |
| 1. Developing a positive identity as a voice hearer |  |  |  |  |  |
| 1. Gaining new social/occupational skills to be used outside the group |  |  |  |  |  |
| 1. Being part of a larger social/political movement |  |  |  |  |  |

1. Do you think there are any other potential advantages for service users attending HVGs?

|  |
| --- |

Please indicate how concerned you are about the following:

|  | I am unconcerned | - I am mostly unconcerned | - I am neither concerned nor unconcerned | - I am a little concerned | - I am very concerned |
| --- | --- | --- | --- | --- | --- |
| 1. Groups might encourage service users to stop taking their medication |  |  |  |  |  |
| 1. Groups may prompt individuals to be critical/sceptical of services or mental health professionals |  |  |  |  |  |
| 1. Groups might encourage service users to disengage from services |  |  |  |  |  |
| 1. Talking about voices may make an individual’s voices worse |  |  |  |  |  |
| 1. Groups may reinforce individual’s delusional beliefs |  |  |  |  |  |
| 1. I am unaware of how HVGs manage risk |  |  |  |  |  |
| 1. Groups are not sufficiently evidence-based |  |  |  |  |  |
| 1. Groups are anti-psychiatry |  |  |  |  |  |
| 1. Seeing others who are highly distressed may make individuals pessimistic about their own recovery |  |  |  |  |  |
| 1. Groups may just turn into a place for people to complain, which may make individuals feel worse |  |  |  |  |  |

1. Do you have any other concerns?

|  |
| --- |

1. Would these concerns prevent you from referring someone to an HVG?

| - Yes |  | - No |  |
| --- | --- | --- | --- |

1. If so, which concerns:

|  |
| --- |

1. Are there any assurances you would need before referring someone on your caseload to a HVG?

|  |
| --- |

1. Who do you think is best suited to facilitate HVGs?

| - Peers/individuals with lived experience |  | - Mental health professionals |  | - A combination of the two |  |
| --- | --- | --- | --- | --- | --- |

Please indicate how much you think each of the following items are barriers to offering more HVGs within the NHS:

|  | - Strongly Disagree | - Disagree | - Neither agree or disagree | - Agree | - Strongly Agree |
| --- | --- | --- | --- | --- | --- |
| 1. Lack of peer facilitators |  |  |  |  |  |
| 1. Lack of professional facilitators |  |  |  |  |  |
| 1. Time required to train facilitators |  |  |  |  |  |
| 1. Time required to run groups |  |  |  |  |  |
| 1. Finding a suitable time/place for groups to take place |  |  |  |  |  |
| 1. Lack of resources to set up and facilitate online groups |  |  |  |  |  |
| 1. Lack of interest from service users |  |  |  |  |  |
| 1. Lack of knowledge of HVGs |  |  |  |  |  |
| 1. High level of staff turnover |  |  |  |  |  |
| 1. High level of service user turnover/throughput |  |  |  |  |  |
| 1. Lack of money to pay facilitators |  |  |  |  |  |
| 1. Ideological differences between HVGs and mental health services |  |  |  |  |  |

1. Do you think there are any other barriers to implementing HVGs more widely within the NHS?

|  |
| --- |

Do you think any of the following features of groups would increase the likelihood of routinely implementing them into the NHS? (Please refer to the description of HVGs on page 1 for a summary of how groups are run outside of NHS settings)

|  | - Much less likely | - Slightly less likely | - Neutral | - Slightly more likely | - Much more likely |
| --- | --- | --- | --- | --- | --- |
| 1. Having a structured, rather than unstructured, intervention |  |  |  |  |  |
| 1. Time-limited/time-specified group involvement |  |  |  |  |  |
| 1. Professional facilitators |  |  |  |  |  |
| 1. Integrated psychoeducation |  |  |  |  |  |
| 1. Integrated CBT |  |  |  |  |  |
| 1. Integrated DBT |  |  |  |  |  |

1. Are there any other features you think would increase the likelihood of routinely implementing HVGs into the NHS?

|  |
| --- |

1. Do you have any thoughts about risk management in HVGs?

|  |
| --- |

1. Would you be more inclined to refer to somebody to an online HVG as opposed to a face-to-face HVG?

| - Yes |  | - No |  | - Depends on the individual |  |
| --- | --- | --- | --- | --- | --- |

If yes, please explain your answer:

|  |
| --- |

1. Would you have any concerns about someone on your caseload taking part in an online HVG?

|  |
| --- |

1. Would you find it preferable to have access to clinical notes summarising what went on in HVGs?

| - Yes |  | - No |  |
| --- | --- | --- | --- |

1. Do you have any other thoughts about HVGs or their implementation in the NHS that you would like to share?

|  |
| --- |

Thank you for taking the time to complete this survey.
