# Supplementary material for: Staff and voice hearer perspectives on Hearing Voices Groups in the NHS: a mixed-methods cross-sectional survey
Source: Front Psychol. 2025 Jul 4;16:1583370. doi: 10.3389/fpsyg.2025.1583370 (PMC12271199; doi:10.3389/fpsyg.2025.1583370)
Supplement: Supplementary file 2 [file Table_2.docx]

Supplementary Material 2

# Supplementary Material 2: HVG Member Survey

**Hearing Voices Groups in the NHS – HVG Member Survey**

Thank you for taking the time to complete this survey. Your answers to this survey are anonymous. You should feel free to skip any questions that you do not wish to answer and provide only as much detail as feels comfortable for you. Please do not include any personal identifiable information in the free text boxes.

We are interested to hear your opinions on Hearing Voices Groups (HVGs). HVGs are peer support groups where individuals can come together and talk openly about what it’s like to hear voices or have other unusual sensory experiences, such as seeing visions. There are many groups like this in the community and online. However, we would like to know if they should also be included in the NHS and, if so, what they would look like.

1. Please tell us your age __________________________________________________
2. Please tell us your gender ________________________________________________
3. Please tell us your ethnicity ______________________________________________
4. How old were you when you first starting hearing voices? ______________________
5. How many voices do you hear?

| - One |  | - Two – Five |  | - Six – Ten |  | - More than Ten |  |
| --- | --- | --- | --- | --- | --- | --- | --- |

1. If you have received one or more psychiatric diagnoses, please write it here: ______________________________________________________________________
2. Please tell us what type of team you receive support from (if you have one):

| - EDIT |  | - Early Intervention |  | - CMHT |  |
| --- | --- | --- | --- | --- | --- |
| - Home Based Treatment |  | - Residential Care |  | - Other (Please describe) |  |

1. Have you ever attended a hearing voices group?

| - Yes |  | - No |  |
| --- | --- | --- | --- |

1. If yes, where was this group was held?

| - Community |  | - NHS Service |  |
| --- | --- | --- | --- |
| - Online |  | - Unsure |  |

1. What made you want to attend your first hearing voices group?

|  |
| --- |

1. Please tell us about your experience in the hearing voices group. For example, was there anything about the group that you found helpful or unhelpful?

|  |
| --- |

1. Would you be interested in attending a hearing voices group that is run in the NHS?

| - Yes |  | - No |  |
| --- | --- | --- | --- |

1. Please tell us a bit more about why you would or would not want to attend a hearing voices group run in the NHS:

|  |
| --- |

If you were to attend a HVG in the NHS, how important are each of these features?

|  | - Not at all important | - Not very important | Neither important nor unimportant | - A little important | Very important |
| --- | --- | --- | --- | --- | --- |
| 1. To meet other people with similar experiences |  |  |  |  |  |
| 1. To learn about how many other people hear voices |  |  |  |  |  |
| 1. To be able to talk about other mental health experiences (for example mood, visions) |  |  |  |  |  |
| 1. To feel less distressed by my voices |  |  |  |  |  |
| 1. To be able to talk about difficult life experiences |  |  |  |  |  |
| 1. To be able to talk about current events and how they make me feel |  |  |  |  |  |
| 1. To be able to talk about what my voices say |  |  |  |  |  |
| 1. To be able to talk about how my voices make me feel |  |  |  |  |  |
| 1. To understand why I hear voices |  |  |  |  |  |
| 1. For the group to be facilitated by a voice hearer |  |  |  |  |  |
| 1. For the group to be facilitated by a mental health professional |  |  |  |  |  |
| 1. For the group to have a structure and agenda |  |  |  |  |  |
| 1. To understand the potential meaning of voices |  |  |  |  |  |
| 1. To learn practical skills to cope with my voices |  |  |  |  |  |
| 1. To learn practical skills to interact with my voices |  |  |  |  |  |
| 1. For the group to be confidential |  |  |  |  |  |
| 1. To learn different frameworks for explaining voices (e.g., spiritual, cultural, trauma-based) |  |  |  |  |  |
| 1. To learn more about the Hearing Voices Movement |  |  |  |  |  |
| 1. To feel more positive about being a voice hearer |  |  |  |  |  |
| 1. To gain new skills to use outside the group |  |  |  |  |  |
| 1. To be able to meet other people who may have heard voices for longer than me |  |  |  |  |  |

1. Are there other features of HVGs that would be important to you?

|  |
| --- |

If you were to attend a HVG in the NHS, would you be concerned about any of the following:

|  | I am unconcerned | - I am mostly unconcerned | - I am neither concerned nor unconcerned | I am a little concerned | - I am very concerned |
| --- | --- | --- | --- | --- | --- |
| 1. I might be judged by other group members |  |  |  |  |  |
| 1. Talking about my voices might make them worse |  |  |  |  |  |
| 1. My voices forbid me to talk about them or say I will be punished if I talk about them |  |  |  |  |  |
| 1. I’m concerned about meeting new people |  |  |  |  |  |
| 1. I might get sectioned if I talk about certain things |  |  |  |  |  |
| 1. My medication might get increased if I talk about certain things |  |  |  |  |  |
| 1. Social services might get involved if I talk about certain things |  |  |  |  |  |
| 1. Other group members might tell me to stop taking my medication |  |  |  |  |  |
| 1. My care team will find out what I say in the group |  |  |  |  |  |
| 1. I might be expected to talk about things I don’t want to talk about |  |  |  |  |  |
| 1. Other group members won’t understand my experiences |  |  |  |  |  |

1. Do you have any other concerns about attending an HVG in the NHS?

|  |
| --- |

Hearing Voices Groups can take place either face-to-face or online. Please tell us a little bit more about your thoughts on these different types of groups.

In your opinion, some of the benefits of attending an online Hearing Voices Group (for example, on Zoom) would be:

|  | - Strongly Disagree | - Disagree | - Neither Agree nor Disagree | - Agree | - Strongly Agree |
| --- | --- | --- | --- | --- | --- |
| 1. I feel safer/more comfortable at home |  |  |  |  |  |
| 1. My voices feel safer/more comfortable at home |  |  |  |  |  |
| 1. I wouldn’t have to arrange travel |  |  |  |  |  |
| 1. I wouldn’t have to arrange childcare |  |  |  |  |  |
| 1. I would be able to have my camera off |  |  |  |  |  |
| 1. It fits into my schedule more easily |  |  |  |  |  |
| 1. I can have a carer with me in the group |  |  |  |  |  |
| 1. I can write in the chat if I don’t want to speak |  |  |  |  |  |
| 1. I find it easier to connect with other people online |  |  |  |  |  |
| 1. I can do other tasks while attending the group (e.g., eating, answering emails) |  |  |  |  |  |

1. Are there any other benefits to attending an HVG online?

|  |
| --- |

In your opinion, some of the challenges in attending an online HVG would be:

|  | - Strongly Disagree | - Disagree | - Neither Agree nor Disagree | - Agree | - Strongly Agree |
| --- | --- | --- | --- | --- | --- |
| 1. I don’t have a laptop/tablet/phone |  |  |  |  |  |
| 1. I don’t know how to use Zoom/Teams |  |  |  |  |  |
| 1. I don’t feel comfortable having my camera on |  |  |  |  |  |
| 1. I’m concerned about others spying on me |  |  |  |  |  |
| 1. I don’t feel comfortable talking about my voices online |  |  |  |  |  |
| 1. I don’t trust technology |  |  |  |  |  |
| 1. I don’t have a private space to join the group |  |  |  |  |  |
| 1. The group might be less confidential because I don’t know who is in the background |  |  |  |  |  |
| 1. My voices don’t feel comfortable online |  |  |  |  |  |
| 1. I find it harder to connect with other people online |  |  |  |  |  |
| 1. I get more distracted online |  |  |  |  |  |

1. Are there any other challenges to attending a group online?

|  |
| --- |

1. I would prefer to attend an HVG:

| - Face-to-Face |  | - Online (for example, on Zoom) |  | - No preference |  |
| --- | --- | --- | --- | --- | --- |

1. Do you have any other thoughts about Hearing Voices Groups in the NHS that you would like to share?

|  |
| --- |

Thank you for taking the time to complete this survey!
