# Supplementary material for: Staff and voice hearer perspectives on Hearing Voices Groups in the NHS: a mixed-methods cross-sectional survey
Source: Front Psychol. 2025 Jul 4;16:1583370. doi: 10.3389/fpsyg.2025.1583370 (PMC12271199; doi:10.3389/fpsyg.2025.1583370)
Supplement: Supplementary file 3 [file Table_3.docx]

Supplementary Material 3

# NHS Staff Survey Results Presented in Table Format

|  | Very important | A little important | Neither important nor unimportant | Not very important | Not at all important | Data missing |
| --- | --- | --- | --- | --- | --- | --- |
| Connecting with others with similar experiences | 100% | 0% | 0% | 0% | 0% | 0% |
| Reducing shame around voices | 100% | 0% | 0% | 0% | 0% | 0% |
| Reducing stigma | 96% | 4.1% | 0% | 0% | 0% | 0% |
| Normalising voice hearing | 83.7% | 16.3% | 0% | 0% | 0% | 0% |
| Reducing distress | 83.7% | 14.3% | 2% | 0% | 0% | 0% |
| Developing a positive identity as a voice hearer | 83.7% | 10.2% | 4.1% | 0% | 0% | 2% |
| Learning new ways to cope with voices | 80% | 20.4% | 0% | 0% | 0% | 0% |
| Providing opportunities to speak about systemic oppression (e.g., racism, poverty, homophobia) | 65.3% | 28.6% | 4.1% | 2% | 0% | 0% |
| Learning new ways to engage with voices | 63.3% | 30.6% | 4.1% | 2% | 0% | 0% |
| Gaining new social/occupational skills to use outside the group | 61.2% | 30.6% | 6.1% | 0% | 2% | 0% |
| Understanding the potential meaning of voices | 55.1% | 34.7% | 6.1% | 4.1% | 0% | 0% |
| Being part of a larger social/political movement | 51% | 22.4% | 24.5% | 2% | 0% | 0% |
| Providing opportunities to speak about adverse life events | 46.9% | 44.9% | 8.2% | 0% | 0% | 0% |
| Providing alternative understandings of voices beyond those of mental health services (e.g., spiritual, cultural) | 46.9% | 40.8% | 10.2% | 0% | 0% | 2% |
| Understanding the potential origin of voices | 40.8% | 42.9% | 8.2% | 8.2% | 0% | 0% |

Supplementary Table 1. NHS staff perspectives on the benefits of HVGs

|  | Very concerned | A little concerned | Neither concerned nor unconcerned | Mostly unconcerned | Unconcerned | Data missing |
| --- | --- | --- | --- | --- | --- | --- |
| Groups may prompt individuals to be critical/sceptical of services or mental health professionals | 4.1% | 10.2% | 14.3% | 42.9% | 28.6% | 0% |
| I am unaware of how HVGs manage risk | 2% | 40.8% | 22.4% | 24.5% | 10.2% | 0% |
| Groups may just turn into a place for people to complain | 2% | 20.4% | 6.1% | 38.8% | 32.7% | 0% |
| Groups might encourage service users to stop taking their medication | 2% | 14.3% | 18.4% | 30.6% | 34.7% | 0% |
| Seeing others who are highly distressed may make individuals pessimistic about their own recovery | 0% | 22.4% | 20.4% | 40.8% | 16.3% | 0% |
| Groups are not sufficiently evidence-based | 0% | 16.3% | 22.4% | 22.4% | 38.8% | 0% |
| Groups might encourage service users to disengage from services | 0% | 14.3% | 12.2% | 34.7% | 38.8% | 0% |
| Groups may reinforce delusional beliefs | 0% | 10.2% | 18.4% | 34.7% | 36.7% | 0% |
| Talking about voices may make an individual's voices worse | 0% | 8.2% | 12.2% | 24.5% | 55.1% | 0% |
| Groups are anti-psychiatry | 0% | 2.4% | 14.3% | 32.7% | 51% | 0% |

Supplementary Table 2. NHS staff concerns about HVGs

|  | Strongly agree | Agree | Neither agree nor disagree | Disagree | Strongly disagree | Data missing |
| --- | --- | --- | --- | --- | --- | --- |
| Lack of peer facilitators | 22.4% | 44.9% | 20.4% | 10.2% | 2% | 0% |
| Lack of money to pay facilitators | 20.4% | 40.8% | 18.4% | 18.4% | 0% | 2% |
| Finding a suitable time/place for groups to take place | 18.4% | 42.9% | 24.5% | 12.2% | 2% | 0% |
| Lack of resources to set up and facilitate online groups | 18.4% | 28.6% | 26.5% | 22.4% | 2% | 2% |
| Time required to train facilitators | 16.3% | 55.1% | 20.4% | 6.1% | 0% | 2% |
| Time required to run groups | 16.3% | 51% | 16.3% | 16.3% | 0% | 0% |
| Lack of professional facilitators | 16.3% | 51% | 14.3% | 12.2% | 6.1% | 0% |
| Lack of knowledge of HVGs | 16.3% | 49% | 16.3% | 16.3% | 0% | 2% |
| High level of staff turnover | 16.3% | 40.8% | 22.4% | 20.4% | 0% | 0% |
| Ideological differences between HVGs and mental health services | 8.2% | 24.5% | 30.6% | 32.7% | 2% | 2% |
| Lack of interest from service users | 6.1% | 12.2% | 20.4% | 44.9% | 16.3% | 0% |
| High level of service user turnover/throughput | 4.1% | 22% | 30.6% | 40.8% | 0% | 2% |

Supplementary Table 3. NHS staff perceptions on barriers to HVG implementation

|  | Much more likely | Slightly more likely | Neutral | Slightly less likely | Much less likely | Data missing |
| --- | --- | --- | --- | --- | --- | --- |
| Professional facilitators | 35.4% | 43.8% | 14.6% | 6.3% | 0% | 0% |
| Time-limited/time-specified group involvement | 25% | 47.9% | 20.8% | 4.2% | 2.1% | 0% |
| Integrated psychoeducation | 20.8% | 66.7% | 12.5% | 0% | 0% | 0% |
| Having a structured, rather than unstructured, intervention | 14.6% | 70.8% | 12.5% | 2.1% | 0% | 0% |
| Integrated DBT | 8.3% | 54.2% | 27.1% | 8.3% | 2.1% | 0% |
| Integrated CBT | 6.3% | 58.3% | 29.2% | 2.1% | 4.2% | 0% |

Supplementary Table 4. NHS staff perceptions on factors that would facilitate HVG implementation in the NHS
