# Supplementary material for: Staff and voice hearer perspectives on Hearing Voices Groups in the NHS: a mixed-methods cross-sectional survey
Source: Front Psychol. 2025 Jul 4;16:1583370. doi: 10.3389/fpsyg.2025.1583370 (PMC12271199; doi:10.3389/fpsyg.2025.1583370)
Supplement: Supplementary file 4 [file Table_4.docx]

Supplementary Material 4

# HVG Member Survey Results Presented in Table Format

|  | Very important | A little important | Neither important nor unimportant | Not very important | Not at all important | Data missing |
| --- | --- | --- | --- | --- | --- | --- |
| To meet other people with similar experiences | 92.3% | 3.8% | 0% | 0% | 3.8% | 0% |
| To be able to talk openly about other mental health experiences (mood, visions) | 92.3% | 3.8% | 0% | 0% | 3.8% | 0% |
| For the group to be confidential | 88.5% | 3.8% | 3.8% | 0% | 3.8% | 0% |
| To be able to talk about how my voices make me feel | 69.2% | 23.1% | 3.8% | 0% | 3.8% | 0% |
| To feel less distressed by my voices | 69.2% | 15.4% | 11.5% | 0% | 3.8% | 0% |
| To learn practical skills to cope with my voices | 65.4% | 30.1% | 0% | 0% | 3.8% | 0% |
| To be able to talk about difficult life experiences | 65.4% | 11.5% | 11.5% | 3.8% | 7.7% | 0% |
| To learn different frameworks for explaining voices (e.g., spiritual, cultural, trauma-based) | 61.5% | 23.1% | 7.7% | 3.8% | 3.8% | 0% |
| To feel more positive about being a voice hearer | 61.5% | 23.1% | 7.7% | 0% | 7.7% | 0% |
| To be able to talk openly about what my voices say | 61.5% | 19.2% | 11.5% | 3.8% | 3.8% | 0% |
| To understand why I hear voices | 61.5% | 19.2% | 11.5% | 3.8% | 3.8% | 0% |
| To gain new skills to use outside the group | 57.7% | 23.1% | 11.5% | 3.8% | 3.8% | 0% |
| For the group to be facilitated by a voice hearer | 57.7% | 15.4% | 23.1% | 0% | 3.8% | 0% |
| To be able to talk about current events and how they make me feel | 53.8% | 30.8% | 7.7% | 3.8% | 3.8% | 0% |
| To learn practical skills to interact with my voices | 53.8% | 23.1% | 11.5% | 3.8% | 7.7% | 0% |
| To be able to meet other people who may have heard voices for longer than me | 50% | 26.9% | 7.7% | 7.7% | 7.7% | 0% |
| To understand the potential meaning of voices | 46.2% | 26.9% | 19.2% | 3.8% | 3.8% | 0% |
| To learn about how many other people hear voices | 38.5% | 38.5% | 7.7% | 7.7% | 7.7% | 0% |
| To learn more about the Hearing Voices Movement | 38.5% | 30.8% | 19.2% | 3.8% | 7.7% | 0% |
| For the group to have a structure and agenda | 19.2% | 42.3% | 11.5% | 12.9% | 7.7% | 0% |
| For the group to be facilitated by a mental health professional | 15.4% | 23.1% | 23.1% | 15.4% | 23.1% | 0% |

Supplementary Table 5. HVG members’ endorsement of important features of HVGs in the NHS

|  | Very concerned | A little concerned | Neither concerned nor unconcerned | Mostly unconcerned | Unconcerned | Data missing |
| --- | --- | --- | --- | --- | --- | --- |
| My medication might get increased if I talk about certain things | 34.6% | 19.2% | 7.7% | 19.2% | 23.1% | 0% |
| Social services might get involved if I talk about certain things | 34.6% | 15.4% | 7.7% | 23.1% | 23.1% | 0% |
| My care team will find out what I said in the group | 30.8% | 15.4% | 7.7% | 19.2% | 30.8% | 0% |
| I might get sectioned if I talk about certain things | 26.9% | 30.8% | 7.7% | 15.4% | 23.1% | 0% |
| My voices forbid me to talk about them or say I will be punished if I talk about them | 19.2% | 26.9% | 3.8% | 15.4% | 38.5% | 0% |
| I might be expected to talk about things I don’t want to talk about | 15.4% | 26.9% | 15.4% | 15.4% | 30.8% | 0% |
| I might be judged by other group members | 7.7% | 30.8% | 23.1% | 26.9% | 15.4% | 0% |
| Other group members might tell me to stop taking my medication | 3.8% | 19.2% | 7.7% | 15.4% | 57.7% | 0% |
| I’m concerned about meeting new people | 0% | 50% | 19.2% | 3.8% | 30.8% | 0% |
| Talking about my voices might make my voices worse | 0% | 30.8% | 3.8% | 38.5% | 30.8% | 0% |
| Other group members won’t understand my experiences | 0% | 19.2% | 19.2% | 30.8% | 34.6% | 0% |

Supplementary Table 6. HVG members’ concerns about HVGs in the NHS

|  | Strongly agree | Agree | Neither agree nor disagree | Disagree | Strongly disagree | Data missing |
| --- | --- | --- | --- | --- | --- | --- |
| I feel safer/more comfortable at home | 51.9% | 14.8% | 14.8% | 14.8% | 3.7% | 0% |
| I wouldn’t have to arrange travel | 48.1% | 37% | 7.4% | 3.7% | 3.7% | 0% |
| It fits into my schedule more easily | 40.7% | 29.6% | 18.5% | 7.4% | 3.7% | 0% |
| My voices feel safer/more comfortable at home | 37% | 11.1% | 33.3% | 11.1% | 7.4% | 0% |
| I can do other tasks while attending the group (e.g., eating, answering emails) | 33.3% | 14.8% | 18.5% | 7.4% | 22.2% | 3.7% |
| I find it easier to connect with other people online | 33.3% | 14.8% | 18.5% | 3.7% | 25.9% | 3.7% |
| I can write in the chat if I don’t want to speak | 29.6% | 33.3% | 22.2% | 7.4% | 7.4% | 0% |
| I would be able to have my camera off | 22.2% | 22.2% | 33.3% | 11.1% | 11.1% | 0% |
| I can have a carer with me in the group | 18.5% | 0% | 44.4% | 22.2% | 14.8% | 0% |
| I wouldn’t have to arrange childcare | 7.4% | 0% | 74.1% | 3.7% | 14.8% | 0% |

Supplementary Table 7. HVG members’ perceptions of the benefits of online HVGs

|  | Strongly agree | Agree | Neither agree nor disagree | Disagree | Strongly disagree | Data missing |
| --- | --- | --- | --- | --- | --- | --- |
| I don't have a laptop/tablet/phone | 30.7% | 0% | 19.2% | 7.7% | 42.3% | 0% |
| I find it harder to connect with other people online | 15.4% | 11.5% | 23.1% | 23.1% | 26.9% | 0% |
| The group might be less confidential because I don't know who is in the background | 11.5% | 30.7% | 15.4% | 15.4% | 26.9% | 0% |
| I am concerned about others spying on me | 11.5% | 19.2% | 26.9% | 11.5% | 30.7% | 0% |
| I get more distracted online | 7.7% | 15.4% | 15.4% | 23.1% | 38.5% | 0% |
| I don't trust technology | 7.7% | 11.5% | 19.2% | 15.4% | 46.2% | 0% |
| I don't know how to use Zoom/Teams | 7.7% | 7.7% | 15.4% | 19.2% | 50% | 0% |
| I don’t feel comfortable having my camera on | 3.8% | 26.9% | 19.2% | 11.5% | 38.5% | 0% |
| My voices don't feel comfortable online | 3.8% | 11.5% | 30.7% | 19.2% | 34.6% | 0% |
| I don't have a private space to join the group | 3.8% | 11.5% | 23.1% | 11.5% | 50% | 0% |
| I don't feel comfortable talking about my voices online | 3.8% | 11.5% | 19.2% | 23.1% | 42.3% | 0% |

Supplementary Table 8. HVG members’ perceptions of the challenges of online HVGs
